# Criticisms on the Treatment of the Late President

**Published:** 1882-01

**Authors:** 


### CRITICISMS ON THE TREATMENT OF THE LATE PRESIDENT.

Many criticisms are being made on the case of the late President; among the most prominent are four which appeared in the *North American Review*, for December, 1881. In this *Review*, Drs. John Ashurst, Jr., J. Marion Sims, and John T. Hodgen, write as apologists for the attendants of the late President; Dr. W. A. Hammond appears as a *critic*. From these gentlemen we shall now quote:

Dr. Ashurst concludes: 1. The diagnosis was erroneous, "but I confess I do not see how the mistake could have been avoided." 2. There was no evidence to show that he suffered from malaria. 3. No evidence of pyæmia (i. e. "septicæmia"). 4. President did not receive sufficient nourishment; cause, weak digestion. 5. The wound was not necessarily fatal, but was made so in this case by his age, weak digestion, probably diseased liver and kidneys, the care and anxiety of his official responsibilities. In conclusion: "Looking at the whole case, from the beginning to the end, I do not see that the treatment could have been altered in any way to the advantage of the illustrious patient; nothing was done that should have been omitted, and nothing was left undone that could possibly have been of benefit."

Dr. Sims concludes: 1. "The President died of septic infection of the blood. It was blood-poisoning, whether called pyæmia or septicæmia." 2. "A correct diagnosis is essential to successful treatment. It is the basis of all treatment. But we may diagnose accurately, and yet our patient dies when the wound is mortal. We may fail to diagnose correctly, and our patient dies for the same reason, that the wound is mortal. In a mortal wound the diagnosis true or false, is powerless to save life. So it was with the President; his wound was mortal, whether correctly diagnosed or not. * * * Without the wound of the vertebræ, it would have been impossible for him to die; with it, it was impossible for him to live."

Dr. Hodgen concludes: 1. That it is remarkable how little morphia was used "to relieve pain and secure rest."(!!) 2. Quotes approvingly Dr. Bliss's statement "that quinine had been given in tonic doses much of the time, and when periodicity was noticeable, sedative doses were administered." 3. "I can find no reason for adverse criticism of any part of the management of this case. I do not find that anything was done, either at the examination or in the treatment to hasten death."

We leave these *scientists* to reconcile their contradictions.

Dr. Hammond commences his criticisms with quotations showing that the wound was not necessarily mortal (agreeing here with Dr. Ashurst), and cruelly quotes Dr. Hamilton as an authority to prove this point. Dr. Hammond says: "While the President's wound was a serious one, there was not a single feature, or combination of features about it which rendered death inevitable." 2. Quotations are made to prove that surgery demands prompt measures and careful treatment of gunshot wounds, especially in the first cleansing, dressing, etc.; that all foreign bodies be removed, etc. This was neglected. These quotations show conclusively that in the opinion of surgical writers, this wound was neither mortal, nor well treated *in the begin-*

*ning at least.* 3. The vigorous antiphlogistic treatment reduced his powers. 4. There has been no proof adduced that the splenic artery was ruptured by the ball, nor was hemorrhage the cause of death.

Such are the contradictory conclusions of these *masters in medical science!* We may well ask what did cause President Garfield's death? One says it was not the wound; another denies that malaria or septicæmia were factors.

In the *Annals of Anatomy and Surgery*, for November, 1881, Dr. Lewis S. Pilcher analyzes the case closely and clearly, concluding that the ball did *not deflect*, but

"That the ball from the moment of its entering into the body till it finally rested behind the pancreas, pursued a straight, undeviating course," and hence could have been found and extracted. He says: "There is one lesson which this case teaches which is especially illustrated in the report made by the principal attending surgeon—namely, the danger of making a diagnosis. It is evident that having made a diagnosis of a lesion of minor importance, all its mutations were by him interpreted in the light of that diagnosis and the significance of the profound symptoms which the case presented failed to be appreciated by him. A candid confession that the data were insufficient, and the retaining of the mind in a judicial state throughout, would have saved medical science from the opprobrium which has been cast upon it by the revelations of the autopsy in this case. The possibility of a preconceived opinion —yclept diagnosis—to warp the judgment, explains how it was possible for bulletins announcing uninterrupted progress toward recovery to be issued when the condition was really one of uninterrupted emaciation and septic infection; for the physicians to announce that the symptoms showed improvement, while the Secretary of State telegraphs that the symptoms are of the gravest character and the strength failing; and for declarations that the patient is convalescent, when he is at the point of death from intense septicæmia."

Yet another critic appears in the person of a Dr. Turnipseed (*Med. Record*, Dec. 3d, 1881). Says Dr. Turnipseed;

"The whole question is, in a nut-shell: Is this modern surgery? Can all the resources of modern surgery do no more than was done for an unfortunate man shot down as poor President Garfield was? If this be true, we should invoke the manes of the great French surgeon, Velpeau, and cause to be echoed and re echoed throughout this broad land his dying words to the young physicians who surrounded his bedside: '*Travaillez! Travaillez toujours!*'"

He too thinks it unlikely that the ball could have deflected and believes that Dr. Bliss may have made "one or more false passages" in his careless probing.

"It appears, however, whether the probing did it or not, the pus burrowed just in the direction in which he probed." * * * *

"*Prognosis.*—Presidont Garfield's case was not necessarily fatal. He naturally was a man of large, fine physique; never was dissapated; was in good health at the time of injury, and the promptness with which his system responded to all efforts used by his surgeons to bring about reaction was favorable, in the abstract, to the highest degree." * * *

"The very healthy condition of the patient at the outset, and the great struggle made by his constitution for life, was clearly demonstrated by the bony union of one or more of the fractured ribs, and the effort at reparation revealed by the autopsy.

"Some may ask what would have become of the fractured vertebræ and spiculæ of bone in the vicinity of the track at various points.

"As a full answer would extend this article, already too long, I refer them to the literature of the profession, to be convinced of what nature, diet, medicines, and the knife have accomplished in wounds when the lesions were fully as grave."

As a fit conclusion to this discussion, we quote Dr. Wm. Hunt (formerly of the University of Pennsylvania), who charges that Drs. Hammond and Sims are ignorant of the "anatomical fact, that arteries are found empty of blood after death." These gentlemen having charged that the blood found in the President's abdomen, at the autopsy, was pushed out of the splenic artery by the injection of *Zinc chlor.*

Dr. Hunt says:

"Have these gentlemen forgotten the very elementary principles and facts of their training as to the blood-vessels?" * * * *

"Those who have spent years in the sculleries of dissecting rooms and in the rooms themselves know practically that nothing is pushed out from *arteries* by injections, for a very plain reason: that there is nothing to push out."

In accounting for the *Zinc chlor.* found in the abdomen at the autopsy, Dr. Hunt says:

"How did the watery solution go in the President's case? It filled the arterial system, and escaped where? Just where the blood had escaped *before death, it escaped after death.*"

To this attack, Dr. Hammond promptly replies (*Med. Record*, Dec. 10), thus:

"To answer Dr. Hunt's questions categorically, I have only to say:

"1. That I was very early in my student-life taught that the name artery is a misnomer.

"2. That I was taught that the ancients from the time of Galen knew perfectly well that the arteries did not contain air, and that consequently we did not have to wait for Harvey to tell us this.

"And I will add:

"That I was also taught that the idea that all the arteries are found empty after death is erroneous, and that my own observation has convinced me of the fact.

"If I had got my teaching entirely from the dissecting-rooms and their 'sculleries,' I might have been brought to believe that the whole arterial system became completely empty at the death of the individual; whereas it is a matter of fact that it does nothing of the kind. The arteries contract after death, but the process does not begin immediately. They are therefore of smaller size than during life, and hence they contain less blood; but, as a rule, with the exception of the large vessels near the heart, they are full, and this is especially true of the splenic artery. This artery is, as my friend knows better than I do, remarkably tortuous, its coats are thicker than those of any other artery in the body, and it is the largest branch of the cœliac axis. Hence (particularly from the thickness of its coats), its calibre is not materially diminished after death, and it always contains a large proportion of blood. * * *

"But I do not wish to have the questions raised by Dr. Hunt depend upon my own opinion. I have, therefore, to 'hurl' at him two or three authorities.

"Muller, speaking of the contractility of the arteries, as exhibited after death, says:

"'They are, in fact, for the most part not quite empty, but contain as much blood as they are able to admit in their contracted state.'

"And again: 'It is not rare to find blood in the arteries after death.'

Relative to the peculiarities of the splenic artery, I quote from Kolliker, as follows:

"'The size of the splenic arteries is very considerable in proportion to that of the organ, and so also the thickness of their coats is worthy of notice. * * * In the mammalia generally the splenic artery is proportionally smaller than in men. This possibly depends only upon the more considerable contraction of the vessels at their death. Wintringham finds that the thickness of the arterial coats is greater than that of the aorta above the giving off of the renal arteries, to which it bears the ratio of 1 to 0.762. He also states that they will sustain a pressure of forty-one pounds.'

"Dissecting-room subjects are not suitable for studies of the kind in question. Days, even months, sometimes elapse before they are examined. As soon as they are obtained a preservative fluid is injected into the vessels, and then perhaps a further injection for display purposes is introduced. In the case of General Garfield the chloride of zinc solution was injected at so short a period after death that there was not time for the arteries to contract to any considerable extent.

"If my distinguished friend will only call to mind his anatomical knowledge, derived from his extensive hospital experience and the numerous post-mortem examinations he has made, and forget for the moment that which has come to him from the dissecting rooms, I think he will agree with me that there was some warrant for my assertion that the extravasated blood in the President's case *might* have been poured out from the splenic artery after death. And I think also that, upon mature reflection, he will recall his opinion that the extraordinary and unscientific proceeding of injecting with chloride of zinc a body upon which the most important post-mortem examination of our time was to be performed was a good thing."

